# The Critical Role of The Piezo1/β‐catenin/ATF4 Axis on The Stemness of Gli1^+^ BMSCs During Simulated Microgravity‐Induced Bone Loss

**DOI:** 10.1002/advs.202303375

**Published:** 2023-09-27

**Authors:** Yuxiang Hu, Hongtao Tian, Wei Chen, Yunlu Liu, Yulin Cao, Hongxin Pei, Chaochang Ming, Cunqing Shan, Xihui Chen, Zhipeng Dai, Shuhua Yang, Zengwu Shao, Shenghui Lan, Yong Liu, Wei Tong

**Affiliations:** ^1^ Department of Orthopedics, Union Hospital, Tongji Medical College Huazhong University of Science and Technology Wuhan Hubei 430022 China; ^2^ Department of Orthopedics The Third Hospital of Hebei Medical University Shi Jiazhuang Hebei 050051 China; ^3^ NHC Key Laboratory of Intelligent Orthopedic Equipment The Third Hospital of Hebei Medical University Shi Jiazhuang Hebei 050051 China; ^4^ Department of Orthopedics, The Central Hospital of Wuhan, Tongji Medical College Huazhong University of Science and Technology Wuhan Hubei 430014 China; ^5^ Department of Orthopedics Wuhan Orthopedic Hospital, Wuhan Puai Hospital Wuhan Hubei 430033 China; ^6^ Department of Orthopedics, Henan Provincial People's Hospital Zhengzhou University People's Hospital Zhengzhou Henan 450003 China; ^7^ Department of Orthopedics, The Eighth People’s Hospital Jiangsu University Shanghai 200235 China; ^8^ Department of Orthopedics, Xuhui Branch of The Sixth People's Hospital Shanghai Jiao Tong University Shanghai 200233 China

**Keywords:** Piezo1, simulated microgravity, disuse osteoporosis, Gli1+ BMSCs, Wnt/β‐catenin/ATF4

## Abstract

Disuse osteoporosis is characterized by decreased bone mass caused by abnormal mechanical stimulation of bone. Piezo1 is a major mechanosensitive ion channel in bone homeostasis. However, whether intervening in the action of Piezo1 can rescue disuse osteoporosis remains unresolved. In this study, a commonly‐used hindlimb‐unloading model is employed to simulate microgravity. By single‐cell RNA sequencing, bone marrow‐derived mesenchymal stem cells (BMSCs) are the most downregulated cell cluster, and coincidentally, Piezo1 expression is mostly enriched in those cells, and is substantially downregulated by unloading. Importantly, activation of Piezo1 by systemically‐introducing yoda1 mimics the effects of mechanical stimulation and thus ameliorates bone loss under simulated microgravity. Mechanistically, Piezo1 activation promotes the proliferation and osteogenic differentiation of Gli1^+^ BMSCs by activating the β‐catenin and its target gene activating transcription factor 4 (ATF4). Inhibiting β‐catenin expression substantially attenuates the effect of yoda1 on bone loss, possibly due to inhibited proliferation and osteogenic differentiation capability of Gli1^+^ BMSCs mediated by ATF4. Lastly, Piezo1 activation also slightly alleviates the osteoporosis of OVX and aged mice. In conclusion, impaired function of Piezo1 in BMSCs leads to insufficient bone formation especially caused by abnormal mechanical stimuli, and is thus a potential therapeutic target for osteoporosis.

## Graphical abstract

Activation of Piezo1 by yoda1 promotes the proliferation and osteogenic differentiation of Gli1^+^ BMSCs through the β‐catenin/ATF4 signaling pathway, which greatly alleviates bone loss under simulated microgravity. This research identifies a novel mechanism for the development of osteoporosis under simulated microgravity and offers a potential therapeutic target for osteoporosis, especially caused by weightlessness or disuse.

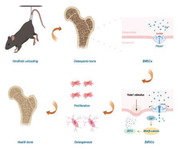

## 1 Introduction

Disuse osteoporosis (DOP) is a worldwide clinical disease characterized by decreased bone mass and increased bone fragility as a result of decreased skeletal mechanical stimulation. It is often secondary to long‐term immobilization or bed rest, long‐term living in a microgravity environment, or muscle activity disorders caused by mental illness or injury.^[^ ^1^, ^2^ ^]^ The pathogenesis is very complex, and no pharmacologic therapy is currently available for the treatment of DOP, which seriously endangers public health. However, the regulatory mechanism between mechanics and bone metabolism is still unclear, and consequently exploring how mechanical loading regulates bone remodeling is expected to lead to novel therapeutic strategies for DOP.

In bone tissue, it has been reported that self‐renewal and cell fate determination of bone marrow‐derived mesenchymal stem cells (BMSCs) and their derived osteoblastic‐lineage cells are extremely sensitive to changes in the extracellular environment and related factors, including extracellular matrix elasticity,^[^ ^3^ ^]^ 3D scaffolds,^[^ ^4^ ^]^ and especially stress, strain, cyclic stretching, fluid shear stress and other mechanical stimuli.^[^ ^5^, ^6^, ^7^, ^8^ ^]^ Multiple mechanical stimuli can modulate the proliferation and differentiation of BMSCs.^[^ ^9^, ^10^ ^]^ For example, fluid shear stress (FSS) increases the expression of osteogenic genes in MSCs to promote osteogenic differentiation.^[^ ^11^, ^12^ ^]^ Similarly, short‐term fluid flow stimulation also promotes the expression of Cox2, OPN and Runx2 at an early stage of osteogenesis in MSCs, while long‐term fluid flow stimulation promotes the formation of collagen and matrix at a late stage of osteogenesis, affecting MSC differentiation.^[^ ^13^ ^]^ However, how mechanical signals are transformed into biological signals by BMSCs, and thus lead to abnormal bone remodeling remains largely unknown.

The discovery of Piezo channels (Piezo1 and Piezo2) has opened a new area to understand how cells respond to the mechanical properties of the microenvironment.^[^ ^14^, ^15^ ^]^ Piezo1 protein is the first truly mechanosensitive ion channel discovered recently, which can directly transfer various mechanical stimuli, including static pressure, shear stress, and membrane stretching, into bioelectrical signals to regulate biological functions of a wide variety of cells,^[^ ^16^, ^17^, ^18^, ^19^, ^20^ ^]^ including those involved in bone remodeling.^[^ ^21^ ^]^ Constitutive knockout of Piezo1 in osteoblasts causes a decrease in bone volume, accelerates bone resorption, and results in fragility fractures,^[^ ^22^ ^]^ while activation of the Piezo1 channel promotes expression of the Wnt1 protein in bone cells to increase bone mass.^[^ ^23^, ^24^ ^]^ However, the role of Piezo1 in mesenchymal stem cells and its downstream mechanism have not yet been fully delineated under abnormal biomechanical conditions.

In this study, we used a hindlimb‐unloading mouse model to simulate the bioeffect of microgravity i*n vivo*. We analyzed cell clusters in the bone marrow of both the ground and unloading mice by single‐cell RNA sequencing and found that the expression of Piezo1 was notably decreased under a simulated microgravity environment. Next, administration of yoda1, a Piezo1 agonist, substantially rescued the bone loss induced by simulated microgravity, possibly due to upregulation of the β‐catenin/ activating transcription factor 4 (ATF4) signaling pathway in Gli1^+^ BMSCs, by enhancing the proliferation and osteogenic differentiation of those cells. Finally, activation of Piezo1 also slightly alleviated the bone loss in OVX and aged mice, which was not as obvious as that of the hindlimb‐unloading model. Collectively, our findings revealed a novel mechanism of osteoporosis caused by simulated microgravity, regulated by Piezo1/β‐catenin/ATF4 in BMSCs, which may be a potential therapeutic target for osteoporosis, especially caused by weightlessness or disuse.

## 2 Results

### 2.1 Piezo1 Expression by Bone Marrow MSCs was Significantly Down‐Regulated by Simulated Microgravity

**Figure 1:**
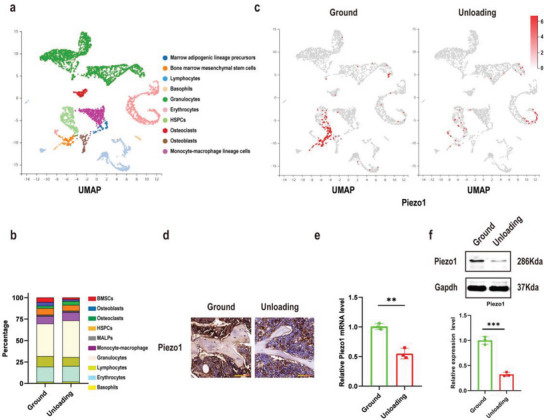
Piezo1 expression of bone marrow‐derived mesenchymal stem cells (BMSCs) was significantly down regulated by simulated microgravity. a) UMAP plot pooled from 10 cell clusters isolated from bone marrow cells of ground and unloading mice by single‐cell transcriptomics. The name of each cell cluster is shown on the right. b) Stacked bars showing the percentage of each cell type in ground and unloading mice bone marrow cells, based on the UMAP distribution. c) The cells were colored on the UMAP plot according to Piezo1 gene expression levels in ground and unloading mouse bone marrow cells. d) Immunohistochemical staining of Piezo1 in femoral sections from ground and unloading mice. Scale bar = 50 µm. e) RT‐PCR analysis of Piezo1 mRNA expression in BMSCs isolated from ground and unloading mice. Mean ± SD, *n* = 3 in each group. **p < 0.01. f) Western blot analysis of Piezo1 protein expression level in BMSCs isolated from ground and unloading mice. Mean ± SD, *n* = 3 in each group. ***p < 0.001.

In this study, a commonly‐used hindlimb‐unloading model was employed to simulate microgravity effects on bone in vivo.^[^ ^25^ ^]^ To understand the changes in the bone marrow microenvironment after four weeks of hindlimb unloading, we first analyzed mouse bone marrow cells from ground and unloading mice by single‐cell RNA sequencing (scRNA‐seq). After rigorous cell filtration, 13758 cells were eventually collected from bone marrow in ground and unloading mice for subsequent analysis. By analyzing the expression of marker genes (Table S2, Supporting Information), we identified four groups of immune cells: including granulocytes, basophils, lymphocytes and monocyte‐macrophage lineage cells. Two groups of osteoblastic lineage cells were also identified, which are bone marrow mesenchymal stem cells (BMSCs) and osteoblasts, followed by osteoclasts, adipogenic lineage cells and hematopoietic lineage cells (**Figure** **1**a). Interestingly, the proportion of BMSCs was notably reduced in unloading mice (4.1%) compared with ground mice (9.6%) (Figure 1b), indicating that a decrease in the number of BMSCs is an important cause of bone loss induced by simulated microgravity. We next analyzed the expression pattern of Piezo1 and found that Piezo1 was highly expressed in BMSCs in ground mice, and was most significantly downregulated after four weeks of unloading (Figure 1c). By contrast, the expression of Piezo2 showed very litter difference between ground and unloading mice (Figure S1A and B, Supporting Information). To further verify the changes of Piezo expression in bone marrow, femurs from ground and unloading mice were collected for immunohistochemical (IHC) staining. In line with scRNA‐seq data, the expression of Piezo1 was much higher in the ground mice (Figure 1d), while Piezo2 expression showed no difference compared with unloading mice (Figure S1C, Supporting Information). Further, mRNA and total protein of bone marrow cells from ground and unloading mice were extracted for RT‐PCR and WB analysis. RT‐PCR proved that Piezo1 mRNA levels were lower in unloading mice than in ground mice (Figure 1e), while the expression of Piezo2 was not significantly changed (Figure S1D, Supporting Information). Meanwhile, the protein expression of Piezo1 was also decreased in the unloading mice (Figure 1f). These results suggested that the decreased number of BMSCs and decreased expression of Piezo1 in these cells were closely related to disuse osteoporosis caused by simulated microgravity.

### 2.2 Activation of Piezo1 Promoted Bone Formation to Rescue Bone Loss under Simulated Microgravity

**Figure 2:**
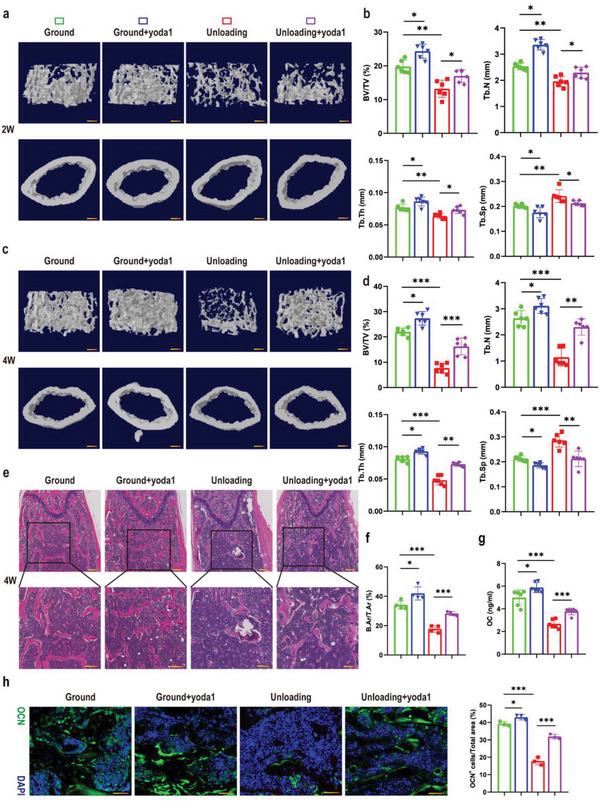
Activation of Piezo1 rescued bone loss under simulated microgravity. a) Representative 3D trabecular architecture and cortical bone images based on micro‐CT reconstruction of the distal femurs at week 2 post‐hindlimb unloading. Scale bar = 500 µm. b) Comparative analysis of the bone parameters including trabecular bone volume fraction (BV/TV), trabecular number (TB. N), trabecular thickness (TB.TH) and trabecular separation (TB. SP) from the distal femurs in the indicated mice at week 2. Mean ± SD, *n* = 6 in each group. *p < 0.05, **p < 0.01, ***p < 0.001. c) Representative 3D trabecular architecture and cortical bone images based on micro‐CT reconstruction in the distal femurs at week 4 post‐hindlimb unloading. Scale bar = 500 µm. d) Comparative analysis of the bone parameters including trabecular bone volume fraction (BV/TV), trabecular number (TB.N), trabecular thickness (TB.TH) and trabecular separation (TB.SP) from the distal femurs in the indicated mice at week 4. Mean ± SD, *n* = 6 in each group. *p < 0.05, **p < 0.01, ***p < 0.001. e) Representative H&E staining images of distal femurs in the indicated mice at week 4. Magnified images of the boxed areas are shown in the panel below. Scale bar = 500 µm (upper image); 100 µm (lower image). f) Quantitative analysis of B.Ar/T.Ar of distal femurs at week 4. Mean ± SD, *n* = 4 in each group. *p < 0.05, ***p < 0.001. B.Ar = bone area; T.Ar = total area; g) Serum OC levels in the indicated mice analyzed by ELISA at week 4. Mean ± SD, *n* = 6 in each group. *p < 0.05, ***p < 0.001. h) Representative immunofluorescent images showing OCN (green) on sections of distal femurs at week 4 post‐hindlimb unloading. Scale bar = 50 µm. The percentage of OCN‐positive cells was calculated. Mean ± SD, *n* = 3 in each group. *p < 0.05, ***p < 0.001. OCN = osteocalcin.

Based on the correlation between Piezo1 and bone loss caused by simulated microgravity, a Piezo1 channel‐specific small molecule agonist (yoda1)^[^ ^26^ ^]^ was used in this hindlimb‐unloading mouse model. We performed micro‐CT analysis to evaluate the trabecular and cortical bone morphology and parameters (BV/TV, TB.N, TB.TH, TB.SP) of distal femurs in the ground, ground + yoda1, unloading, and unloading + yoda1 groups of mice. As seen from the results, mice showed significant trabecular bone loss and decreased cortical bone thickness after two or four weeks of hindlimb unloading compared to the control group in which the mice were fed on the ground. Notably, when the unloading mice were injected with yoda1, bone loss was significantly reduced. In addition, when ground‐fed mice were injected with the same dose of yoda1, there was an increase in bone mass (**Figure** 2a and c).

**Figure 3:**
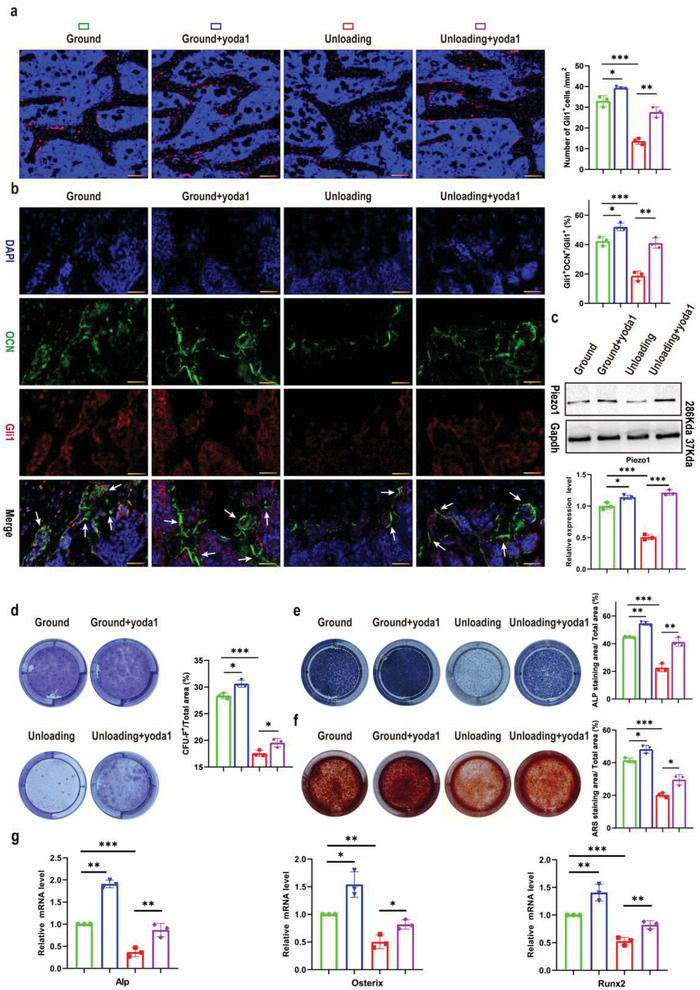
Yoda1 promoted the proliferation and osteogenic differentiation of Gli1^+^ BMSCs both in vivo and in vitro. a) Confocal images showing tdTomato^+^ BMSCs (red) on sections of distal femurs at week 4 post‐hindlimb unloading. Scale bar = 50 µm. Numbers of tdTomato^+^ BMSCs were calculated in the indicated mice. Mean ± SD, *n* = 3 in each group. *p < 0.05, **p < 0.01, ***p < 0.001. b) Representative co‐staining of OCN (green) and Gli1 (red) on sections of distal femurs at 4 weeks post‐hindlimb unloading, counterstained by DAPI (blue). Scale bar = 50 µm. Percentage of Gli1^+^; OCN^+^ over Gli1^+^ was calculated. Mean ± SD, *n* = 3 in each group. *p < 0.05, **p < 0.01, ***p < 0.001. c) Western blot analysis of Piezo1 protein expression levels in the indicated Gli1^+^ BMSCs. Mean ± SD, *n* = 3 in each group. *p < 0.05, ***p < 0.001. d) Gli1^+^ BMSCs were isolated from bone marrow cells of ground and unloading mice and treated with or without yoda1 (2 µM) for 7 days. Clonogenicity of each group was measured by CFU‐F assay. Mean ± SD, n = 3 in each group. *p < 0.05, ***p < 0.001. e, f) Gli1^+^ BMSCs were isolated from bone marrow cells of ground and unloading mice and cultured in osteogenic medium for 14 days with or without yoda1 (2 µm). On day 14, CFU‐OB was evaluated by alkaline phosphatase (top panel) and alizarin red staining (bottom panel). Mean ± SD, *n* = 3 in each group. *p < 0.05, **p < 0.01, ***p < 0.001. g) RT‐PCR analysis of osteogenic marker gene expression levels in the indicated Gli1^+^ BMSCs. Mean ± SD, *n* = 3 in each group. *p < 0.05, **p < 0.01, ***p < 0.001. OCN = osteocalcin.

Regarding bone parameters, the bone volume fraction (BV/TV), trabecular number (TB.N) and trabecular thickness (TB.TH) all decreased significantly, whereas trabecular separation (TB.SP) was accordingly increased in the unloading group compared to the ground group (Figure 2b and d). Interestingly, in the unloading + yoda1 group, the mean BV/TV, TB. N and TB.TH increased 110%, 98% and 44%, respectively, compared to the unloading group at four weeks, so that it was almost identical to the control group that were fed on the ground. In addition the TB.SP showed a 27% reduction (Figure 2d). H&E staining of the femurs further revealed the therapeutic effect of yoda1 on simulated microgravity‐induced bone mass loss (Figure 2e). The bone area / total area in the distal metaphyses of the femurs in the unloading group was significantly reduced compared to the ground group. However, administration of yoda1 not only increased bone area in the ground mice, but more importantly, bone area was significantly increased in the unloading + yoda1 group compared to the unloading group (Figure 2f). Interestingly, we found that yoda1 attenuated the bone loss induced by unloading, mainly by increasing trabecular number and thickness, suggesting that bone formation played an important role in this process. Therefore, to further understand the mechanism of Piezo1 on bone remodeling, we assayed two typical serum markers of bone formation and bone resorption, osteocalcin (OC) and C‐terminal telopeptide of type I collagen (CTX). The results showed that the serum level of CTX in the unloading group was slightly increased and the OC level was dramatically lower in the unloading group, compared with the ground group. However, yoda1 exhibited a significant upregulation of OC and a slight reduction in CTX after unloading (Figure 2g; Figure S2A, Supporting Information). Moreover, we performed immunofluorescence staining of OCN as well as TRAP staining. TRAP staining of the bone sections showed that, compared to the unloading group, the numbers of osteoclasts were slightly lower in the unloading + yoda1 group (Figure S2B, Supporting Information). The osteoclast surface of the bone sections (Oc.S/BS) was increased in the unloading group, while the surface area of osteoclasts on the bone surface was reduced by yoda1 (Figure S2C, Supporting Information). Immunofluorescence staining of OCN suggested that in the unloading group, the number of OCN‐positive cells in the bone marrow was dramatically reduced compared to the ground group, but was greatly increased in the unloading + yoda1 group (**Figure** **3**h). These results suggested that bone formation was significantly inhibited under simulated microgravity conditions, but this was attenuated by Piezo1 activation.

### 2.3 Yoda1 Promoted the Proliferation and Osteogenic Differentiation of Gli1^+^ BMSCs In Vivo and In Vitro

The activity and osteogenic differentiation of MSCs play an important role in bone formation. As we and others have proved that Gli1^+^ stem cells contribute greatly to osteoblastic lineage cells in the murine skeleton,^[^ ^27^, ^28^ ^]^ we generated Gli1‐CreER/Tomato mice for lineage tracing in vivo and confirmed their stemness by CD44 co‐staining (Figure S3A, Supporting Information). As shown in Figure 3a, yoda1 treatment not only increased the number of Gli1 Td^+^ cells in ground mice, but also significantly rescued Gli1 Td^+^ cells in bone marrow caused by hindlimb unloading. To further confirm the role of yoda1 in the osteogenic differentiation of Gli1^+^ cells, we performed immunofluorescence staining. The results showed that the positive expression of OCN was notably reduced in Gli1^+^ cells in the unloading mice, compared to the ground, while yoda1 treatment significantly rescued this effect (Figure 3b). Next, to validate our in vivo data, BMSCs were extracted from 8‐week‐old ground and unloading mice, and we used fluorescence‐activated cell sorting (FACS) to isolate Gli1^+^ BMSCs (Figure S3B, Supporting Information). Interestingly, we found that the expression of Piezo1 was notably decreased in Gli1^+^ BMSCs from unloading mice, but was significantly elevated after yoda1 treatment (Figure 3c). We then evaluated the effect of yoda1 on formation of fibroblast (CFU‐F) and osteoblast (CFU‐OB) colonies by Gli1^+^ BMSCs in vitro. According to the results of crystal violet staining, hindlimb unloading decreased the numbers of CFU‐F by as compared to the ground group, however, the numbers of CFU‐F of ground group and unloading group were increased 8% and 11%, respectively, after treatment with yoda1 (Figure 3d). In accordance with CFU‐OB counts, the numbers of CFU‐OB stained for alkaline phosphatase and alizarin red were also significantly decreased under simulated microgravity condition compared to the ground group, but were obviously elevated after treatment with yoda1 (Figure 3e and f). Additionally, the gene expression levels of osteoblastic markers (Alp, Runx2 and Osterix) of Gli1^+^ BMSCs were also downregulated in the unloading group compared to the ground group, but were greatly enhanced by yoda1 treatment (Figure 3g). Taken together, our results indicated that Piezo1 activation promoted the potential colony‐forming ability and osteogenic differentiation of Gli1^+^ BMSCs in the bone marrow, thereby significantly alleviating the bone loss caused by simulated microgravity.

### 2.4 Piezo1 Acted through Ca^2+^ Influx to Regulate the Expression of β‐Catenin and ATF4 in Gli1^+^ BMSCs

**Figure 4:**
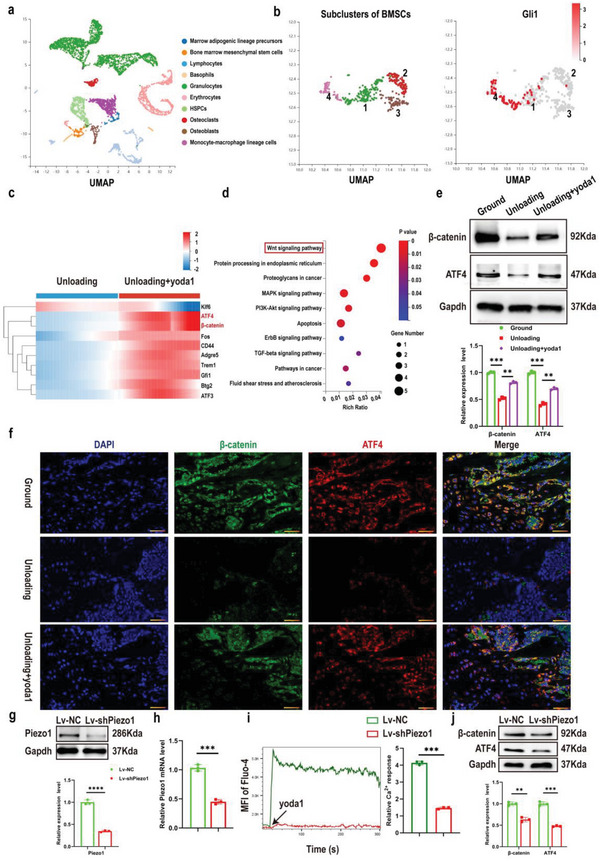
Expression of both β‐catenin and its downstream target gene ATF4 was increased in Gli1^+^ BMSCs after Piezo1 activation. a) Colored UMAP plot created using pooled data from 10 cell clusters from bone marrow‐derived cells isolated from unloading and unloading + yoda1 mice by single‐cell transcriptomics. The name of each cell cluster is shown on the right. b) Among the four subclusters of BMSCs, the expression level of Gli1 was higher in subclusters 1 and 4 compared to subclusters 2 and 3. c) Heatmap generated from scRNA‐seq depicting the differentially‐expressed genes (DEGs) in Gli1^+^BMSCs between unloading and unloading + yoda1 groups. d) KEGG pathway analysis of DEGs. e) Western blot analysis of β‐catenin and ATF4 protein expression levels in the indicated Gli1^+^ BMSCs. Mean ± SD, *n* = 3 in each group. **p < 0.01, ***p < 0.001. f) Representative β‐catenin (green) and ATF4 (red) co‐immunostaining images of distal femurs at week 4 post‐hindlimb unloading. Scale bar = 50 µm. g) Western blot analysis of Piezo1 protein level in Gli1^+^ BMSCs after transfection with NC or Lv‐shPiezo1 for 36 h. Mean ± SD, *n* = 3 in each group. ****p < 0.0001. h) RT‐PCR analysis of Piezo1 mRNA in Gli1^+^ BMSCs after transfection with NC or Lv‐shPiezo1 for 36 h. Mean ± SD, *n* = 3 in each group. ***p < 0.001. i) Representative images showing calcium influx of Gli1^+^ BMSCs stimulated by yoda1 (10 µM) changed over time (left). Cells were transfected with Lv‐NC or Lv‐shPiezo1 for 36 h. The changes in fluorescent intensity of Gli1^+^ BMSCs were quantified (right). Mean ± SD, *n* = 3 in each group. ***p < 0.001. j) Western blot analysis of β‐catenin and ATF4 protein expression levels when Piezo1 was knocked down. Mean ± SD, *n* = 3 in each group. **p < 0.01, ***p < 0.001.

To fully understand the potential mechanism of Piezo1 activation in Gli1^+^ BMSCs, single‐cell RNA sequencing of bone marrow in the unloading and unloading + yoda1 groups was performed as previously described (**Figure** **4**a). Further analysis of BMSCs divided them into four clusters, among which Gli1 was expressed at a higher level in clusters 1 and 4 than clusters 2 and 3 (Figure 4b), marking a clear separation between Gli1^+^ (clusters 1 and 4) and Gli1^−^ (clusters 2 and 3) clusters. Compared with the unloading group, GO and KEGG analysis identified significantly‐elevated genes in Gli1^+^ BMSCs of the unloading + yoda1 groups (Figure S4A, Supporting Information), including β‐catenin and ATF4, shown as a heatmap (Figure 4c). KEGG pathway analysis revealed that these differentially‐expressed genes (DEGs) were enriched in the Wnt signaling pathway (Figure 4d).

The Wnt signaling pathway plays an essential role in regulating bone homeostasis.^[^ ^29^, ^30^ ^]^ Beta‐catenin and ATF4 are important downstream effectors of the Wnt signaling pathway, regulating cell proliferation and differentiation, and promoting bone formation.^[^ ^31^, ^32^, ^33^, ^34^ ^]^ Similarly, GO analysis also showed that these DEGs were highly correlated with the positive regulation of ossification, cell proliferation and osteoblast differentiation (Figure S4B, Supporting Information). Therefore, we further investigated the expression of these key factors in Wnt signaling pathways after yoda1 treatment. WB showed that, compared to the ground group, β‐catenin and ATF4 were both decreased in Gli1^+^ BMSCs isolated from the unloading group, while yoda1 treatment in vitro significantly increased the expression of β‐catenin and ATF4 (Figure 4e). Moreover, immunofluorescence staining also revealed that treatment with yoda1 increased total β‐catenin and ATF4 expression in the bone marrow area (Figure 4f). As Piezo1 is a calcium (Ca^2+^) permeable channel and Ca^2+^ signaling regulates Yap1 and Wnt/β‐catenin activities,^[^ ^24^, ^35^ ^]^ we then determined whether Piezo1 regulates the Wnt/β‐catenin signaling pathway through Ca^2+^ influx. We employed Gadolinium (Gd^3+^), a potent calcium channel blocker including Piezos,^[^ ^36^ ^]^ to block Ca^2+^ influx in Gli1^+^ BMSCs. Interestingly, treating Gli1^+^ BMSCs with yoda1 resulted in rapid calcium influx and increased β‐catenin expression, while this promotional effect induced by yoda1 was significantly suppressed by Gd^3+^ (Figure S4C and D, Supporting Information). Consistent with this, loss of Piezo1 by Lentivirus (Lv) infection also suppressed yoda1‐evoked Ca^2+^ entry into Gli1^+^ BMSCs as well as reducing β‐catenin and ATF4 expression levels (Figure 4g–j). Taken together, our findings strongly suggest that β‐catenin and ATF4 are the key target genes that regulate the proliferation and differentiation of Gli1^+^ BMSCs activated by Piezo1 through Ca^2+^ influx.

### 2.5 Activation of Piezo1 Alleviated the Bone Loss under Simulated Microgravity through Wnt/ β‐Catenin Signaling

**Figure 5:**
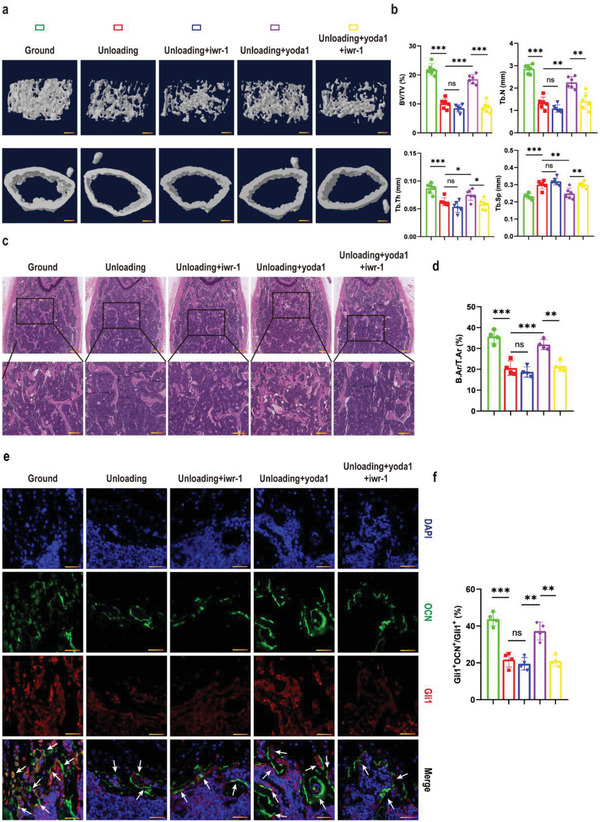
Activation of Piezo1 alleviated bone loss under simulated microgravity by acting through Wnt/β‐catenin signaling pathway. a) Representative 3D trabecular architecture and cortical bone images based on micro‐CT reconstruction of the distal femurs at week 4 in the indicated mice. Scale bar = 500 µm. b) Comparative analysis of the bone parameters BV/TV, TB.N, TB.TH and TB.SP from the distal femurs in the indicated mice at week 4. Mean ± SD, *n* = 6 in each group. *p < 0.05, **p < 0.01, ***p < 0.001, ns = no significant difference. c) Representative H&E staining images of distal femurs in the indicated mice in week 4. Magnified images of the boxed areas are shown in the panel below. Scale bar = 500 µm (upper image); 100 µm (lower image). d) Quantitative analysis of B.Ar/T.Ar of distal femurs in the indicated mice at weeks 4. Mean ± SD, *n* = 4 in each group. **p < 0.01, ***p < 0.001, ns = no significant difference. e) Representative immunofluorescent images of distal femurs at week 4 post‐hindlimb unloading in the indicated mice, immunostained with OCN (green) and Gli1 (red) antibodies and counterstained with DAPI (blue). Scale bar = 50 µm. f) Percentage of Gli1^+^; OCN^+^ over Gli1^+^ was calculated. Mean ± SD, n = 3 in each group. **p < 0.01, ***p < 0.001, ns = no significant difference. B.Ar = bone area; T.Ar = total area. OCN = osteocalcin.

In order to elucidate the involved signaling pathway in vivo, we used IWR‐1, which stabilizes the destruction complex member Axin2, to promote the degradation of β‐catenin.^[^ ^37^ ^]^ IWR‐1 was intraperitoneally injected into unloading mice followed by yoda1. 3D reconstruction of micro‐CT analysis revealed that the unloading mice showed notably lower bone mass. BV/TV, TB.N and TB.TH all decreased significantly, coupled with increased TB.SP. Importantly, yoda1 treatment significantly alleviated the bone loss under simulated microgravity, with a higher BV/TV, TB.N and TB.TH. However, the rescue effects of yoda1 were completely abolished by IWR‐1. The BV/TV, TB. N and TB.TH were decreased 44%, 42% and 23% respectively compared to the yoda1‐treated group (**Figure** 5a and b). Consistent with the micro‐CT results, H&E staining further confirmed the suppressing effects of IWR‐1 on yoda1. As shown in Figure 5c and d, yoda1 significantly increased the bone area in the distal metaphysis of the femurs as expected, but was notably suppressed by IWR‐1. In addition, immunofluorescence staining also showed that administration of yoda1 could not increase the positive expression of OCN in Gli1^+^ cells in the bone marrow of IWR‐1‐treated mice (Figure 5e and f). These data suggested that the Wnt/β‐catenin signaling pathway inhibitor, IWR‐1, significantly blocked the bone volume‐enhancing effects of Piezo1 activation.

### 2.6 Piezo1 Promoted the Proliferation and Osteogenic Differentiation of Gli1^+^ BMSCs Mediated by ATF4

**Figure 6:**
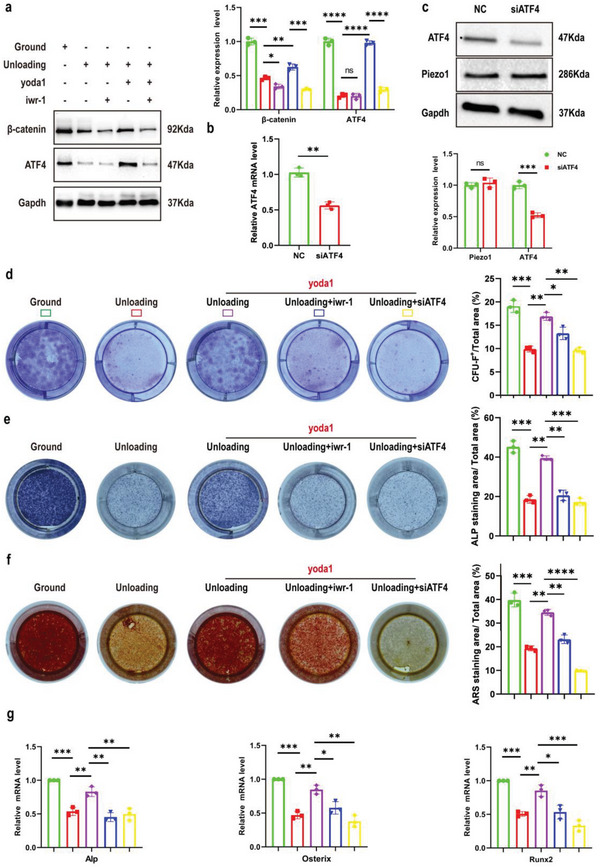
The critical role of Piezo1/β‐catenin in the function of Gli1^+^ BMSCs is mainly mediated by ATF4 under simulated microgravity. a) Western blot analysis of β‐catenin and ATF4 proteins in the indicated Gli1^+^ BMSCs. Mean ± SD, *n* = 3 in each group. *p < 0.05, **p < 0.01, ***p < 0.001, ****p < 0.0001. b) RT‐PCR analysis of ATF4 mRNA in Gli1^+^ BMSCs after transfection with NC siRNA or ATF4 siRNA for 48 h. Mean ± SD, *n* = 3 in each group. **p < 0.01. c) Western blot analysis of Piezo1 and ATF4 protein levels in Gli1^+^ BMSCs after transfection with NC siRNA or ATF4 siRNA for 48 h. Mean ± SD, *n* = 3 in each group. ns = no significant difference, ***p < 0.001. d) Gli1^+^ BMSCs were isolated from bone marrow‐derived cells in ground and unloading mice, treated with IWR‐1 (10 µm) or transfected with ATF4 siRNA, and then treated with or without yoda1 (2 µM) for 7 days. Clonogenicity of each group was measured by CFU‐F assay. Mean ± SD, *n* = 3 in each group. *p < 0.05, ***p < 0.001. e, f) Gli1^+^ BMSCs were isolated from bone marrow‐derived cells in ground and unloading mice, treated with IWR‐1 (10 µm) or transfected with ATF4 siRNA, and cultured in osteogenic medium for 14 days with or without yoda1 (2 µM). On day 14, CFU‐OB formation was measured by alkaline phosphatase (e) and alizarin red staining (f). Mean ± SD, *n* = 3 in each group. *p < 0.05, **p < 0.01, ***p < 0.001. g) RT‐PCR analysis of osteogenic marker gene expression levels in the indicated Gli1^+^ BMSCs. Mean ± SD, n = 3 in each group. *p < 0.05, **p < 0.01, ***p < 0.001.

To verify the downstream effector of Wnt/β‐catenin signaling, we performed western blot analysis and found that IWR‐1 completely eliminated the expression of ATF4 induced by yoda1 (**Figure** **6**a), indicating that yoda1 promoted the expression of ATF4 through Wnt/β‐catenin signaling. To further determine the role of ATF4 in regulating the proliferation and osteogenic differentiation of Gli1^+^ BMSCs, we isolated Gli1^+^ BMSCs as before and knocked down the expression of ATF4 in these cells. Results showed that the expression of ATF4 was significantly decreased by ATF4 siRNA compared to NC siRNA, but we found that the expression of Piezo1 was not affected when ATF4 was inhibited (Figure 6b and c). Next, we explored the effects of IWR‐1 and ATF4 on fibroblast (CFU‐F) and osteoblast (CFU‐OB) colony formation by Gli1^+^ BMSCs. The results showed that IWR‐1 and ATF4 siRNA both significantly inhibited the effect of yoda1 in promoting the formation of fibroblastic colonies and ALP^+^ or ARS^+^ osteoblastic colonies by Gli1^+^ BMSCs (Figure 6d–f). Furthermore, yoda1‐induced expression of osteoblastic markers (Alp, Osterix and Runx2) was significantly inhibited by IWR‐1 or ATF4 siRNA (Fig. 6g). These results suggested that yoda1 promoted the clonogenicity and osteogenic differentiation of Gli1^+^ BMSCs by increasing ATF4 expression through the Wnt/β‐catenin signaling pathway. Taken together, these results suggest that yoda1 activated the Piezo1/β‐catenin/ATF4 axis of Gli1^+^ BMSCs to promote osteogenesis under simulated microgravity, thereby alleviating bone loss caused by simulated microgravity.

### 2.7 Activation of Piezo1 Protected against Bone Loss in OVX and Aging Mice

**Figure 7:**
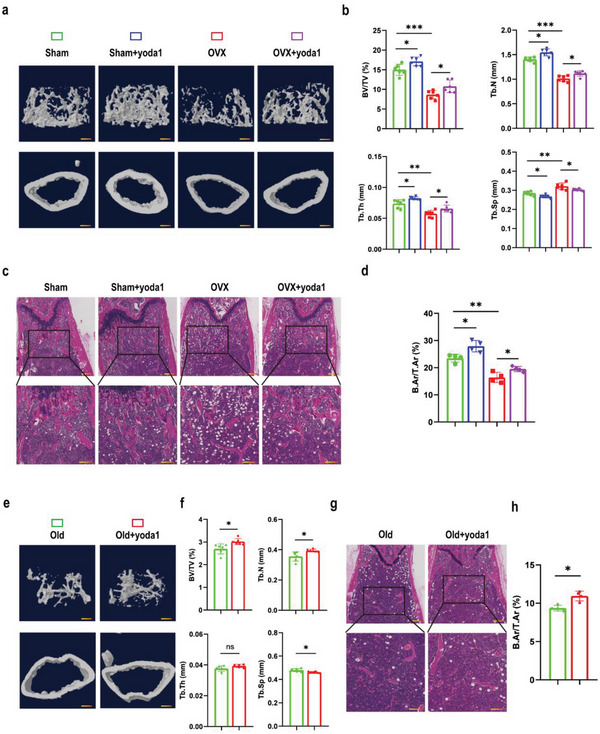
Activation of Piezo1 slightly but significantly rescued bone loss in OVX and aged mouse models. a, e) Representative 3D trabecular architecture and cortical bone images based on micro‐CT reconstruction of the distal femurs at week 4 after the OVX (a) and aged (e) groups of mice indicated after the administration of yoda1. Scale bar = 500 µm. b, f) Comparative analysis of the bone parameters BV/TV, TB.N, TB.TH and TB.SP in the distal femurs from the OVX (n = 6 in each group) (b) and aged (*n* = 6 in each group) (f) groups of mice indicated at week 4. *p < 0.05, **p < 0.01, ***p < 0.001, ns = no significant difference. c, g) Representative H&E staining images of distal femurs from the OVX (n = 4 in each group) (c) and aged (g) (*n* = 4 in each group) groups of mice indicated at week 4. Boxed areas are shown magnified in the panel below. Scale bar = 500 µm (upper image); 100 µm (lower image). d, h) Quantitative analysis of B.Ar/T.Ar ratios in distal femurs from the OVX (d) and aged (h) groups of mice indicated at week 4. Mean ± SD, n = 4 in each group. *p < 0.05, **p < 0.01. B.Ar = bone area; T.Ar = total area.

In the process of exploring the effect of yoda1 on bone loss in unloading mice, we also found that yoda1 slightly improved bone mass in ground mice, indicating that activation of Piezo1 without a mechanical stimulus could also affect bone remodeling. Therefore, we next explored the effect of yoda1 on post‐menopausal and aging‐related osteoporosis. OVX mice were generated to simulate post‐menopausal osteoporosis, then intraperitoneally administered yoda1 or saline for four weeks. Figure S5A,B (Supporting Information) showed that compared to the sham group, uterine volume and weight were significantly lower in the OVX mice, which confirmed that the OVX model was successfully generated. Micro‐CT and H&E analysis revealed that the OVX group exhibited a marked bone mass decrease in the metaphysis of the distal femur compared with the sham group, but that intraperitoneal injection of yoda1 to OVX mice slightly rescued the bone loss (**Figure** **7**a, c and d). Bone structural parameters in the metaphysis of the distal femur based on micro‐CT showed that the values of BV/TV, TB.N and TB.TH were significantly decreased in OVX mice with an increase in TB. SP compared to the sham group. However, when OVX mice were treated with yoda1, the mean BV/TV, TB.N and TB.TH were increased by 27%, 18% and 13% respectively, with an 11% reduction of TB.SP (Figure 7b). Similarly, 3D images (Figure 7E) and H&E staining (Figure 7g and h) showed that administration of yoda1 slightly increased the bone mass in aged mice. Yoda1‐treated aged mice exhibited a 12% increase in BV/TV, a 15% increase in TB.N and an 8% reduction in TB.SP (Figure 7f). Interestingly, we found that the expression of Piezo1 was decreased in BMSCs from both OVX and aging mice, but was elevated after yoda1 treatment (Figure S5C, Supporting Information). These results indicated that activation of Piezo1 had a small protective effect against OVX and aging‐induced bone loss.

## 3 Discussion

Mechanical stimulation is widely recognized as an important factor in embryonic bone formation, postnatal bone development, and adult bone maintenance and repair.^[^ ^38^ ^]^ The main cause of DOP is lack of appropriate mechanical stimulation of bone, which leads to an imbalance of bone remodeling homeostasis.^[^ ^10^ ^]^ Recently, piezoelectric ion channels (Piezo1 and Piezo2) have been identified as mechanosensitive ion channels,^[^ ^39^ ^]^ and play crucial roles in a variety of physiological processes. Piezo1 is mainly expressed in non‐sensory tissues and non‐neuronal cells,^[^ ^40^ ^]^ and is able to sense various mechanical stresses, including gravity, shear stress, and membrane stretch. Piezo2 is mainly expressed in sensory tissues, such as dorsal root ganglion (DRG) sensory neurons and Merkel cells,^[^ ^41^ ^]^ and regulates mechanical nociception.^[^ ^42^ ^]^ Previous studies have found that Piezo1 acts as an important regulator of bone remodeling, regulating both osteoblast lineage cells and osteoclasts in osteoporosis.^[^ ^24^, ^43^ ^]^ However, the specific target cells of Piezo1 remain unclear in DOP. Therefore, in our work, we first conducted an in‐depth study of mouse bone marrow cell subsets by scRNA‐seq, and found that Piezo1 was highly expressed in BMSCs in ground mice, but in the unloading mice, the numbers of BMSCs and the expression of Piezo1 in these cells were both significantly decreased, indicating a strong association between Piezo1 and DOP, and BMSCs were the main responders of Piezo1 channel. Next, activation of Piezo1 mimicked the effects of mechanical stimulation to ameliorate bone loss under simulated microgravity. Further mechanistic studies proved that Piezo1 activation promoted the proliferation and osteogenic differentiation of Gli1^+^ BMSCs by activating β‐catenin/ATF4 signaling pathway under simulated microgravity conditions. Taken together, our findings precisely elucidate the cellular and molecular mechanisms by which Piezo1 regulates bone loss in weightlessness and thus provides a new therapeutic strategy for pathological osteoporosis, especially caused by weightlessness or disuse.

Recent studies have shown that Piezo1 plays an important role in MSC differentiation fate determination under various mechanical stimuli.^[^ ^19^, ^43^ ^]^ Hydrostatic pressure (HP) stimulation induces the expression of Piezo1 and bone morphogenetic protein 2 (Bmp2) in UE7T13 cells (human mesenchymal stem cells), and regulates the fate of mesenchymal stem cells by promoting osteoblast differentiation and inhibiting adipocyte differentiation via Piezo1–Bmp2 pathway.^[^ ^44^ ^]^ In addition to HP stimulation, the expression of Piezo1 and osteoblastic marker genes are also increased in BMSCs when subjected to fluid shear stress (FSS), exhibiting stronger Ca^2+^ influx and enhanced osteogenic differentiation. However, these processes are blocked by Cre adenovirus‐mediated deletion of Piezo1 in MSCs.^[^ ^24^ ^]^ Under FSS stimulation, the activation of Piezo1 further induces the phosphorylation of protein kinase B (PKB/AKT) and GSK‐3β, which in turn leads to the nuclear import of β‐catenin, thereby regulating the expression of Runx2.^[^ ^45^ ^]^ In our study, we found that Piezo1 expression significantly decreased in BMSCs in a simulated microgravity environment, thereby showing a lower osteogenic ability. Further activation of Piezo1 up‐regulated the expression of osteogenic transcription factor ATF4 through the Wnt/β‐catenin signaling pathway, thus promoting the osteogenic differentiation of BMSCs. In addition to the osteoblast lineage discussed above, we also found a subtle increase in the activity of osteoclasts under simulated microgravity. Similar to hindlimb unloading, Xiong et al. also found that knockdown of Piezo1 in osteoblasts and osteocytes not only resulted in decreased bone formation but also increased RANKL expression and increased bone resorption.^[^ ^46^ ^]^ However, how Piezo1 regulates osteoclasts or the cross‐talk between osteoblasts and osteoclasts still needs to be resolved in future studies. In summary, our data demonstrate that Piezo1 plays an important role in the regulation and maintenance of mechano‐mediated bone homeostasis. Piezo1 mediates the mechanotransduction of BMSCs, thereby affecting bone formation and bone resorption. However, a tissue‐specific knockout animal model, such as Piezo1 and ATF4 specific deletion in BMSCs using Gli1 Cre‐ER mice is needed to accurately validate the function of Piezo1 in BMSCs in unloading mice.

The Wnt pathway is an important mechanotransduction pathway in osteocytes.^[^ ^47^ ^]^ As a major effector of the canonical Wnt pathway, β‐catenin has been shown to be critical in osteocyte mechanotransduction. Mice with osteocytes deficient in β‐catenin exhibited severe osteopenia, bone fragility, and a markedly impaired response to mechanical loading.^[^ ^48^, ^49^ ^]^ In addition, ATF4 is also mechanistically regulated for terminal osteoblast differentiation and bone formation.^[^ ^50^, ^51^ ^]^ However, the interaction between β‐catenin and ATF4 and the mechanisms involved in the regulation of bone mass under microgravity have not been investigated. Studies have suggested that ATF4 overexpression increases the expression of β‐catenin and forms a β‐catenin–ATF4 protein complex in osteoblasts.^[^ ^34^ ^]^ Our work here demonstrated that β‐catenin and ATF4 were both decreased under simulated microgravity, while yoda1 treatment increased the expression of β‐catenin and ATF4 both in vitro and in vivo. In addition, IWR‐1, which inhibits the Wnt/β‐catenin signaling pathway, blocked the elevation of ATF4, indicating that ATF4 is mainly regulated by Wnt/β‐catenin, in line with previous studies.^[^ ^52^, ^53^ ^]^ Importantly, either blocking the Wnt/β‐catenin signaling pathway or knockdown of ATF4 largely attenuated the osteogenic effect of yoda1 on Gli1^+^ BMSCs in vitro. Inhibition of Wnt/β‐catenin in vivo also blocked the positive regulation of bone mass by Piezo1 activation under simulated microgravity. Together, these data suggested that β‐catenin/ATF4 signaling may be a major regulatory mechanism of Piezo1 channel activation by yoda1 on mechano‐mediated bone homeostasis.

Osteoporosis has many causes and the mechanism is complex. Hormones, aging, and weightlessness are all related to osteoporosis, so it is necessary to precisely target the pathogenesis for intervention, which is also an important reason for the large variation in the efficacy of osteoporosis treatment. In the present study, we found that activation of Piezo1 also slightly promoted osteoblast differentiation (Figure S6A,B, Supporting Information) and inhibited the differentiation and maturation of osteoclasts (Figure S6C,F, Supporting Information) in OVX and aged mice, thereby alleviating bone loss by 14% and 6% respectively. However, activation of Piezo1 caused 41% reduction in bone loss in disuse osteoporosis, suggesting that activation of Piezo1 is more effective in the treatment of disuse osteoporosis than in hormone or aging related osteoporosis. Indeed, astronauts lose more bone mass in a month than postmenopausal women on Earth in a year, with greater declines in bone strength,^[^ ^54^ ^]^ indicating that the molecular mechanisms underlying bone mass loss due to mechanical force deficiency may be different from those responsible for hormone or aging related osteoporosis. However, how activation of Piezo1 regulates bone formation and resorption in OVX and aged mice needs to be further investigated. Nevertheless, our study demonstrates that activation of Piezo1 channel could be considered as a novel target for the treatment of osteoporosis, especially caused by weightlessness or disuse.

## 4 Experimental Section

### Hindlimb unloading mouse model

Eight‐week‐old male C57BL/6J mice were purchased from Beijing SPF Biotechnology Co., Ltd. Rosa‐td Tomato mice were purchased from Jiangsu GemPharmatech Co., Ltd., (Nanjing, China) and Gli1‐CreER Rosa‐td Tomato (Gli1/Tomato) mice were generated by breeding Rosa‐td Tomato mice with Gli1‐CreER mice obtained from Jackson Laboratory (Bar Harbor, ME, USA). To simulate microgravity in vivo, a hindlimb unloading mouse model was created. In brief, the mice were kept in standard cages, suspended at a 30‐degree angle. Of course, this movement ensured the animals free access to food and water. The appearance, eating habits, and tails of the mice were checked every 2 days. In order to induce CreER activity in mice, tamoxifen (75 mg kg^−1^ d^−1^) was injected subcutaneously every day for 5 days starting before hindlimb unloading. Mice in the treatment group were injected intraperitoneally with 5 mmol kg^−1^ yoda1 (SML1558; Sigma, 40 mM dissolved in DMSO then diluted with 5% ethanol) daily for five consecutive days per week until the time of sacrifice. The mice in the Wnt inhibitor group were intraperitoneally injected with 100 µL of 200 µM IWR‐1 (Selleck Chemicals, Houston, TX, USA; S7086) diluted in phosphate‐buffered saline (PBS), four times a week, while the control group received an equal volume of vehicle. After suspension for four weeks, bilateral femurs were collected for subsequent experiments.

### OVX‐induced osteoporosis and aging male mouse models

Eight‐week‐old female C57BL/6J mice were purchased from Beijing SPF Biotechnology Co., Ltd. and randomly divided into four groups: sham + Veh, sham + yoda1, OVX + Veh, OVX + yoda1. A sham operation or bilateral ovariectomy (OVX) was carried out to establish the OVX mouse model. At two weeks of after the operation, the mice in the sham + yoda1 and OVX + yoda1 groups were intraperitoneally injected with 5 mmol kg^−1^ yoda1 on five consecutive days per week. Meanwhile, mice in sham + Veh and OVX + Veh groups were administered an equal volume of saline solution. Mice in each group were weighed twice a week. After four weeks of treatment, the mice were sacrificed, and samples were collected for subsequent experiments.

For the aging mice, twelve‐month‐old male C57BL/6J mice were kept under standard animal feeding conditions (12 h light, 12 h dark cycle, with free access to food and water) until 20 months of age. Six were randomly selected as the control group, and six were used as the yoda1 treatment group.

All animal research related to this study was approved by the Animal Care and Use Committee of Union Hospital, Huazhong University of Science and Technology (Ethical approval number: 3246).

### Micro‐CT analysis

Mouse femur specimens were collected, fixed with 4% paraformaldehyde for 48 h, and then scanned using a SkyScan 1176 high‐resolution microscopic CT imaging system (micro‐CT) at 9 µm resolution, with a 1 mm aluminum filter, 90 kV voltage, and 273 µA current. The original data obtained were used for volume reconstruction and 3D image generation by NRecon reconstruction software and CT‐VOX 3D reconstruction analysis software provided by SkyScan. Bone parameters such as bone volume/tissue volume (BV/TV), bone trabecular number (TB.N), bone trabecular thickness (TB.TH) and trabecular separation (TB.SP) were measured.

### Histology and immunohistochemistry

After performing micro‐CT scanning, the mouse femurs were fixed with 4% paraformaldehyde for 24 h, then decalcified at room temperature with 10% EDTA for 30 days. The specimens were then dehydrated through a series of graded concentrations of ethanol (70% to 100%) and embedded in paraffin. After embedding, the specimens were sectioned at a thickness of 6 µm and in the sagittal direction. Hematoxylin and eosin (H&E), tartrate‐resistant acid phosphatase (TRAP) and immunohistochemical (IHC) staining were performed. The primary antibodies used for immunohistochemistry were: anti‐Piezo1 (1:100, 15939‐1‐AP, Proteintech, Rosemont, IL, USA), anti‐Piezo2 (1:100, 26205‐1‐AP, Proteintech), anti‐TRAP (1:100, BD Biosciences, Franklin Lakes, NJ, USA, 555 025), anti‐OCN (1:100, Santa Cruz Biotechnology, Santa Cruz, CA, SC‐390877). This experiment was conducted by Hubei Baos Biotechnology Co., Ltd., which provided all the reagents except the primary antibodies.

### ScRNA‐seq and analysis

Bone marrow cells were isolated by flushing the long bones with alpha‐ modified minimum essential medium (α‐MEM, Gibco, Carlsbad, CA, USA) on ice and dissociating the marrow into a single‐cell suspension as previously mentioned.^[^ ^55^ ^]^ Briefly, each single‐cell suspension was converted to a barcoded scRNA‐seq library through a sequence of steps including droplet encapsulation, emulsion breakage, mRNA captured bead collection, reverse transcription, cDNA amplification and purification. Indexed sequencing libraries were constructed according to the manufacturer's protocol. The sequencing libraries were quantified using a Qubit ssDNA Assay Kit (Thermo Fisher Scientific, Waltham, MA, USA). The sequencing libraries were sequenced using the DNBSEQTM platform. The R package Seurat (Version 3.0.2) was used for data analysis.^[^ ^56^ ^]^ Briefly, the principal components in the data was first calculated, then screened out the most significant principal components based on the degree of enrichment and *p* value, combined with UMP or tSNE algorithm (https://www.nature.com/articles/nbt.4314) for nonlinear dimensionality reduction analysis, clustering cells into several types. Differentially‐expressed genes (DEGs) were considered statistically significant at a threshold of adjusted *p*‐value set at 0.05. Gene ontology (GO) analysis, and Kyoto Encyclopedia of genes and Genomes (KEGG) enrichment analysis for DEGs were both performed in the Gene Ontology database  (http://www.geneontology.org/), as previously described.^[^ ^57^ ^]^

### Enzyme‐Linked Immunosorbent Assay (ELISA)

To measure serum OC and CTX levels in mice, blood samples were harvested using an anticoagulant collection vessel. The blood was centrifuged at 3,000 rpm for 10 min, and the supernatant was collected and stored at −20°C until measurement. The serum levels of OC and CTX were measured using a commercially available ELISA kit according to the manufacturer's protocol (both from Immunodiagnostic Systems, Ltd.).

### Cell isolation, treatment and transfection

After four weeks of hindlimb unloading, bone marrow mesenchymal stem cells (BMSCs) were isolated from ground and unloading mice by flushing the femurs with α‐MEM and treated the marrow cell suspension with erythrocyte lysate on ice for 10 min. Then cells were centrifuged, plated into T25 flasks and cultured with α‐MEM (Gibco) containing 10% fetal bovine serum (FBS) (Gibco), penicillin (100 U mL^−1^), streptomycin (100 g mL^−1^). All experiments were performed using passage 1 (P1) BMSCs.

Yoda1 was resuspended in 40 mM DMSO and diluted in culture medium to a final concentration of 2 µM. IWR‐1 was added to cells at the indicated concentrations (10 µM), followed by yoda1.

BMSCs were seeded into 6‐well plates at a density of 1 × 10^4^ cells well^−1^ and then transfected with 100 nM ATF4 siRNA by GP‐transfect‐mate for 48 h. The siRNA‐NC served as negative control. Lentivirus (Lv)‐shPiezo1 (GenePharma Co. Ltd., Shanghai, China) at a multiplicity of infection (MOI) of 50 was incubated with the cells for 12 h. The Lv‐NC served as negative control. After 36 h of infection, cells were subjected to puromycin (2.5 µg mL^−1^) selection for 7 days. The efficacy of gene knockdown was confirmed by RT‐PCR and western blotting before subsequent experiments.

### Flow cytometry

Bone‐marrow‐derived cells were isolated as described above. Bone marrow cells were then centrifuged and resuspended in PBS and filtered through a 50‐µm cell strainer. Cells were stained with the fluorescent‐labeled primary antibodies, rabbit anti‐Gli1 polyclonal antibody (abs121671, Absin, 1:100), goat anti‐rabbit FITC (abs20023, Absin, 1:100), or PE rat anti‐mouse CD44 IM7 (553 134, BD, 1:100) for 30 min at 4°C. Samples were washed with PBS and cells were sorted using a BD Biosciences flow cytometer. Data were analyzed using FlowJo 10.6 software.

### Analysis of calcium concentration

Cells were washed twice with Hanks’ balanced salt solution (HBSS), then incubated with 1 µM fluo‐4 AM (Beyotime Institute of Bbiotechnology, Jiangsu, China) for 1 h at 37°C. After washing twice with HBSS, cells were incubated with 1 µM fluo‐4 AM for another 20 min at 37°C. Then, the mean fluorescence intensity (MFI) before and after yoda1 stimulation (10 µM) was measured by flow cytometry (BD Biosciences) and analyzed using FlowJo 10.6 software.

### CFU‐F and CFU‐OB assay

In the CFU‐F assay, sorted Gli1^+^BMSCs were cultured in a 12‐well dish at 0.5×10^6^ cells well^−1^ in α‐MEM containing 15% FBS for 7 days. On day 7, the adherent cells were fixed with paraformaldehyde and stained with crystal violet.

For the CFU‐OB assay, 10^6^ sorted cells well^−1^ were cultured in a 12‐well plate in α‐MEM containing 15% FBS for 7 days. Then osteogenic differentiation medium was added and exchanged every 2–3 days. On day 14, cells were fixed with paraformaldehyde and stained for alkaline phosphatase or alizarin red.

### Culture and differentiation of bone marrow macrophages (BMM) in vitro

Tibias and femurs were isolated from C57BL/6J mice aged 4–6 weeks. To obtain bone marrow macrophages (BMMs), the bone marrow was flushed from the bones and total bone marrow cells were cultured in α‐MEM (Gibco) containing 10% FBS (Gibco), penicillin (100 U mL^−^^1^), streptomycin (100 g mL^−^^1^) and 30 ng mL^−1^ M‐CSF (Peprotech, Rocky Hill, NJ, USA) for 24 h. Then, to induce osteoclast differentiation, BMMs were resuspended in α‐MEM containing 10% FBS, seeded into a 24‐well plate at a density of 10^5^ well^−1^, and cultured with 30 ng mL^−^^1^ M‐CSF and 50 ng mL^−1^ RANKL (Peprotech) for 7 days, together treated with 0, 0.3, 1 or 3 µM yoda1. The medium was changed every 2 days.

### Tartrate‐resistant acid phosphatase staining

Cells cultured in the 24‐well plates were washed twice with PBS, then fixed with paraformaldehyde for 20 min. A tartrate‐resistant acid phosphatase (TRAP) staining kit was used for analysis, as directed by the manufacturer (Beyotime Institute of Bbiotechnology, Jiangsu, China).

### FITC Phalloidin

BMMs were seeded at a density of 1.6×10^5^ cells well^−1^ onto the glass coverslips placed into wells of a 24‐well plate and treated with 0, 0.3, 1 or 3 µM yoda1. After 24 h treatment, the cells attached to the glass coverslips were washed twice with PBS, and fixed with 4% paraformaldehyde for 10 min. Then cells were permeabilized for 5 min with 0.5% Triton X‐100 and washed again. Afterwards, cells were incubated with 200 µL FITC‐labeled cyclopeptide working solution for 30 min at room temperature, protected from light. After washing, the nuclei were stained with 200 µL DAPI solution (concentration 1:100 nM) for approximately 30 s. Coverslips were mounted on slides with anti‐fluorescence quenching sealed tablets and observed under a confocal microscope (Nikon A1; Nikon, Tokyo, Japan).

### Immunofluorescence

Frozen sections were rewarmed to room temperature, and washed twice with PBS. Next, the sections were permeabilized with 3‰Triton X‐100 at room temperature for 15 min, then blocked in 5% bovine serum for 30 min. Sections were incubated with primary antibodies against Gli1 (1:100, Abclonal, Woburn, MA, USA; A14675), OCN (1:100, Boster, Wuhan, China; PB1008), β‐catenin (1:100, BD Biosciences; 610 154), or ATF4 (1:500, Proteintech, 60035‐1‐Ig) overnight at 4°C. The next day, after washing twice with PBS, sections were labeled with secondary antibody for 1 h in the dark, then stained with DAPI for 10 min and imaged under a confocal microscope (Nikon A1).

For cells cultured on slides, after fixation with 4% paraformaldehyde for 20 min at room temperature, cells were stained with a primary antibody against Gli1 (1:100, Abclonal, A14675) or CD44 (1:200, Proteintech, 15675‐1‐AP) overnight at 4°C. Next day the cells were incubated with secondary antibody for 1 h and then counterstained with DAPI for 10 min in the dark. Images were obtained under a confocal microscope (Nikon A1).

### Real‐time polymerase chain reaction (RT‐PCR)

Total RNA was prepared from cells using TRIzol reagent (TaKaRa Bio, Tokyo, Japan) according to the manufacturer's protocol, and cDNA was synthesized by reverse transcription using a high‐capacity cDNA reverse transcription kit (Applied Biosystems, Foster City, CA, USA). A PCR machine was used for RT‐PCR detection. All primer sequences were designed using Primer 5.0 software. Expression levels were normalized to GAPDH which was used as the internal reference. The 2−ΔΔCT method was used to analyze the relative expression levels of each target gene. The primer sequences used in this study were provided in Table S1 (Supporting information).

### Western blot analysis

Total protein was extracted from cells using RIPA buffer (Thermo Fisher Scientific) supplemented with 1% protease inhibitor on ice. Protein concentration was determined by the BCA assay (Sigma‐Aldrich, St Louis, MO, USA). Next, proteins were subjected to sodium dodecyl sulfate polyacrylamide gel electrophoresis (SDS‐PAGE) and transferred to a 0.45 µm polyvinylidene difluoride membrane. The membranes were blocked with 5% skimmed milk. After washing with TBS containing 0.01% Tween 20 (TBST) three times, membranes were then incubated with the following primary antibodies: anti‐Piezo1 (1:1000, Proteintech, 15939‐1‐AP), β‐catenin (1:1000, BD Biosciences, 610 154) or ATF4 (1:1000, Proteintech, 60035‐1‐Ig) overnight at 4°C. Glyceraldehyde 3‐phosphate dehydrogenase (GAPDH, 1:2000, Abcam, Cambridge, MA, USA; ab8245) was used as the internal reference. Next day, secondary antibodies against the species of primary antibodies were added and incubated for 1 h at room temperature. Signals on the membrane were detected by electrochemiluminescence (ECL, Pierce, Rockford, IL, USA).

### Statistical analysis

All statistical analyses and mapping were performed using GraphPad Prism 8 software (GraphPad Software Inc., La Jolla, CA, USA). The summary graphical data were presented as mean values ± standard deviation (SD) of at least three independent experiments. Two groups were compared via the two‐tailed Student's t test. For comparison among three or more groups, data were analyzed by one‐way analysis of variance (ANOVA) with the Bonferroni post hoc test. Values of *p* < 0.05 were considered statistically significant.

## Conflict of Interest

The authors declare no conflict of interest.

## Supporting information



Supporting Information

## Data Availability

Research data are not shared.
