# Supplementary material for: The Critical Role of The Piezo1/β‐catenin/ATF4 Axis on The Stemness of Gli1+ BMSCs During Simulated Microgravity‐Induced Bone Loss
Source: Adv Sci (Weinh). 2023 Sep 27;10(32):2303375. doi: 10.1002/advs.202303375 (PMC10646271; doi:10.1002/advs.202303375)
Supplement: Supplementary file 1 — Supporting Information [file ADVS-10-2303375-s001.pdf]

ADVANCED SCIENCE
Open Access

Supporting Information

for *Adv. Sci.*, DOI 10.1002/advs.202303375

The Critical Role of The Piezo1/β-catenin/ATF4 Axis on The Stemness of Gli1$^+$ BMSCs During Simulated Microgravity-Induced Bone Loss

Yuxiang Hu, Hongtao Tian, Wei Chen, Yunlu Liu, Yulin Cao, Hongxin Pei, Chaochang Ming, Cunqing Shan, Xihui Chen, Zhipeng Dai, Shuhua Yang, Zengwu Shao, Shenghui Lan, Yong Liu* and Wei Tong**

Table S1: Real-time PCR primer sequences used in this study

Gene	Forward primer (5' to 3')	Reverse primer (3' to 5')
Piezo1	GCTTGCTAGAACTTCACG	GTACTCATGCGGGTTG
Piezo2	AGTCTGGAAGCTCAACACCG	AGCCAGATGGTCAGACTTGC
ATF4	GAGCTTCCTGAACAGCGAAGTG	TGGCCACCTCCAGATAGTCATC
Alp	ATCTTTGGTCTGGCTCCCATG	TTTCCCGTTCACCGTCCAC
Runx2	ACGAAAAATTAACGCCAGTCG	TCGGTCTGACGACGCTAAAG
Osterix	AGAGGTTCACTCGCTCTGACGA	TTGCTCAAGTGGTCGCTTCTG
Gapdh	GGTTGTCTCCTGCGACTTCA	TGGTCCAGGGTTTCTTACTCC

Table S2: Marker genes for cell populations used in this study

Cell type	Cell marker
Erythrocytes	Klf1, Gypa, Epor, Hbb-bs
Granulocyte	CD33, S100a8, S100a9
Lymphocytes	Cd79a, Cd79b, Cd19, Vpreb3
Basophils	Prss34
Hematopoietic stem and progenitor cells (HSPCs)	Cd34, Spn, Cd48
Monocyte-macrophage lineage cells	Ly6c2, Cd14, Ms4a4c, Ccr2, Sell
Osteoblasts	Sp7, Runx2, Bglap2, Col1a1
Osteoclasts	Mmp9, Ctsk, Dcstamp, Nfatc1, Acp5
Bone marrow mesenchymal stem cells (BMSCs)	Lepr, Cxcl12, Ng2, Cd44, Angpt1, Nes
Marrow adipogenic lineage precursors	Lpl, Adipoq, Apoe

(MALPs)

Figure S1: Piezo2 expression in mouse bone marrow under simulated microgravity conditions. (A) A UMAP plot pooled from 10 cell clusters isolated from bone marrow-derived cells of ground and unloading mice by single-cell transcriptomics. The name of each cell cluster is defined in the right panel. (B) The cells are colored on the UMAP plot according to Piezo2 gene expression levels in ground and unloading mouse bone marrow-derived cells. (C) Immunohistochemical staining of Piezo2 in femoral sections from ground and unloading mice. Scale bar = 50 μm. (D) RT-PCR analysis of Piezo2 mRNA expression level in bone marrow mesenchymal stem cells isolated from ground and unloading mice. Mean ± SD, n = 3 in each group; ns = no significant difference.

Figure S2: Yoda1 slightly suppressed bone resorption under simulated microgravity conditions. (A) Serum CTX levels in the indicated mice analyzed by ELISA. Mean ± SD, n = 6 in each group. $*p < 0.05$. (B) Representative TRAP staining images of sections of distal femurs at 4 weeks post-hindlimb unloading. TRAP-positive cells in the distal femurs are indicated by orange-colored arrows. Scale bar = 50 μm. (C)

Quantification of osteoclast surface (Oc.S)/bone surface (BS) in the indicated mice. Mean ± SD, n = 4 in each group. *p < 0.05.

Figure S3: Gli1 labeled a subset of BMSCs in bone marrow. (A) Representative immunofluorescence images showing $Gli1^+$ cells (red) co-expressing the BMSC marker CD44 (green). Scale bar = 50 μm. (B) $Gli1^+$ BMSCs were sorted by flow cytometry from ground and unloading mice.

Figure S4: Quantitative transcriptomic and yoda1-induced Ca^{2+} response analysis of $Gli1^+$ BMSCs isolated from unloading or unloading + yoda1 mice. (A) Differentially expressed genes (DEGs) are listed in the unloading and unloading + yoda1 group. (B) GO biological process analysis of DEGs. (C) Representative images showing changes in the calcium influx of $Gli1^+$ BMSCs stimulated by yoda1 (10 μM) over time (left). Cells were incubated with veh or Gd^{3+} (100 nM) for 24 h. The fluorescence intensity changes of $Gli1^+$ BMSCs were quantified (right). Mean ± SD, n = 3 in each group. ***p < 0.001. (D) Western blot analysis of β-catenin protein expression levels in the indicated $Gli1^+$ BMSCs. Mean ± SD, n = 3 in each group. ***p < 0.001.

Figure S5: Uterine morphology, gravimetric and western blot analysis after OVX. (A) Representative images of uterine morphology in the indicated mice. (B) Uterine weight was measured in the indicated mice. Mean ± SD, n = 6 in each group. *p < 0.05, ***p < 0.001, ns = no

significant difference. (C) Western blot analysis of Piezo1 protein expression levels in the indicated BMSCs. Mean ± SD, n = 3 in each group. *$p < 0.05$, **$p < 0.01$, ***$p < 0.001$.

Figure S6: Effects of yoda1 on osteoblast and osteoclast differentiation. (A, B) Representative OCN staining images of sections of distal femurs from mice 4 weeks after the OVX (A) and aged mice (B) after the administration of yoda1. OCN-positive cells in the distal femurs are indicated by red-colored arrows. Scale bar = 50 μm. Quantification of osteoblast surface (Ob.S)/bone surface (BS) from the OVX (n = 4 in each group) and aged (n = 4 in each group) mice indicated at 4 weeks. Mean ± SD, *$p < 0.05$, ***$p < 0.001$. (C, D) Representative TRAP staining images of sections of distal femurs from mice at 4 weeks after OVX (C) and aged mice (D) after the administration of yoda1. TRAP-positive cells in the distal femurs are indicated by orange-colored arrows. Scale bar = 50 μm. Quantification of osteoclast surface (Oc.S)/bone surface (BS) from the OVX (n = 4 in each group) and aged (n = 4 in each group) groups of mice at 4 weeks. Mean ± SD, *$p < 0.05$, **$p < 0.01$. (E) Representative images showing that the formation of multinucleated osteoclasts was inhibited by yoda1 in a dose-dependent manner. Scale bar = 100 μm. (F) Representative immunostaining images showing the actin rings and nuclei, stained with phalloidin-FITC and DAPI, respectively, in the indicated groups. Scale bar = 100 μm. OCN = osteocalcin.

Figure S1

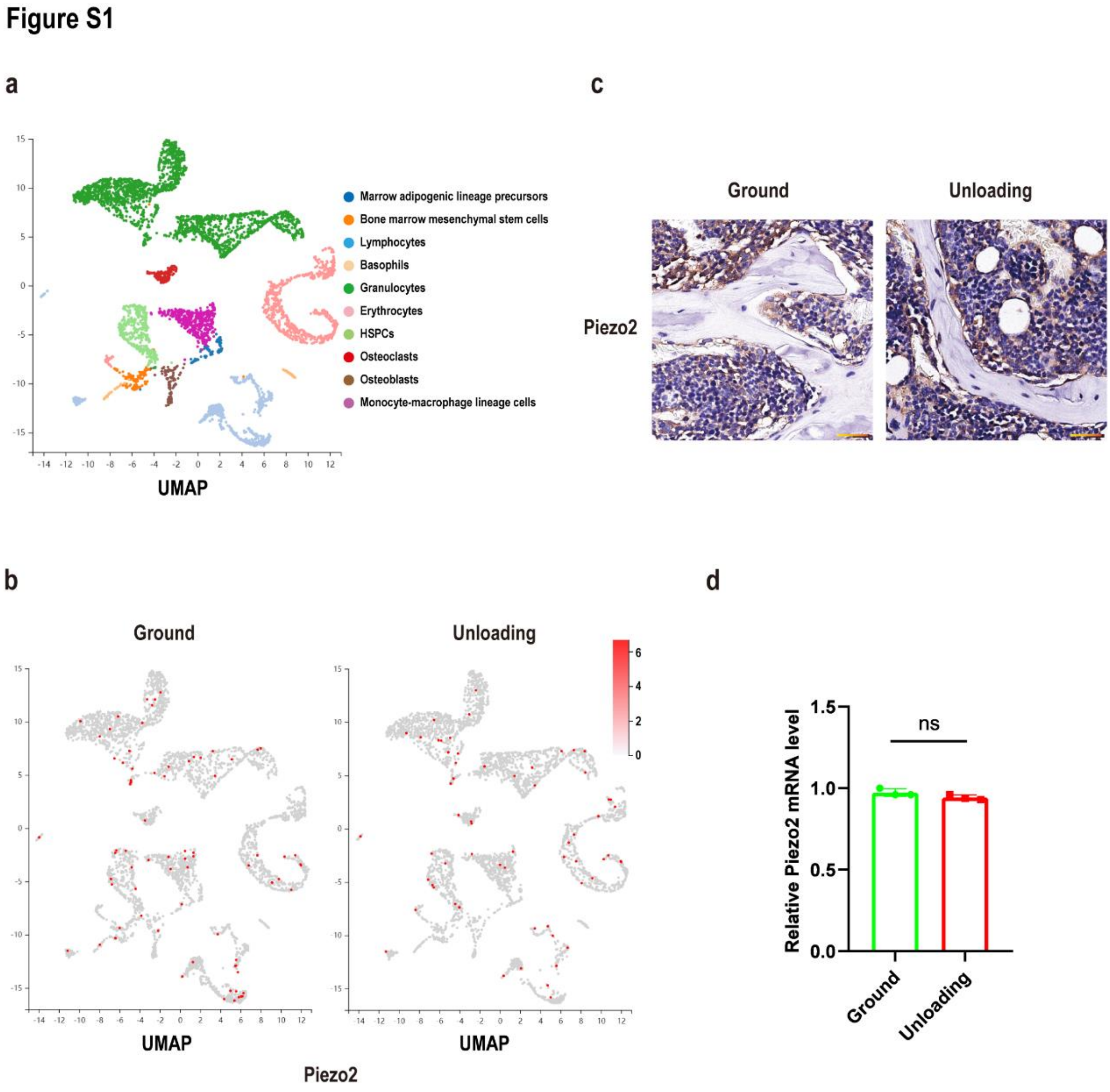
Figure S1
a
Marrow adipogenic lineage precursors
Bone marrow mesenchymal stem cells
Lymphocytes
Basophils
Granulocytes
Erythrocytes
HSPCs
Osteoclasts
Osteoblasts
Monocyte-macrophage lineage cells
UMAP
b
Ground
Unloading
UMAP
UMAP
Piezo2
c
Ground
Unloading
Piezo2
d
Relative Piezo2 mRNA level
ns
Ground
Unloading

Figure S2

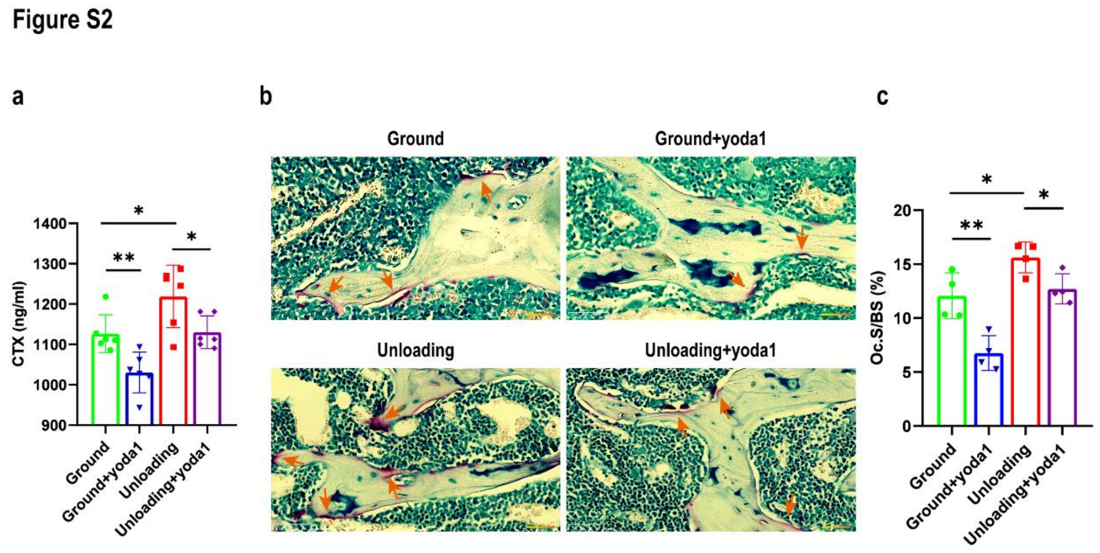

Figure S3

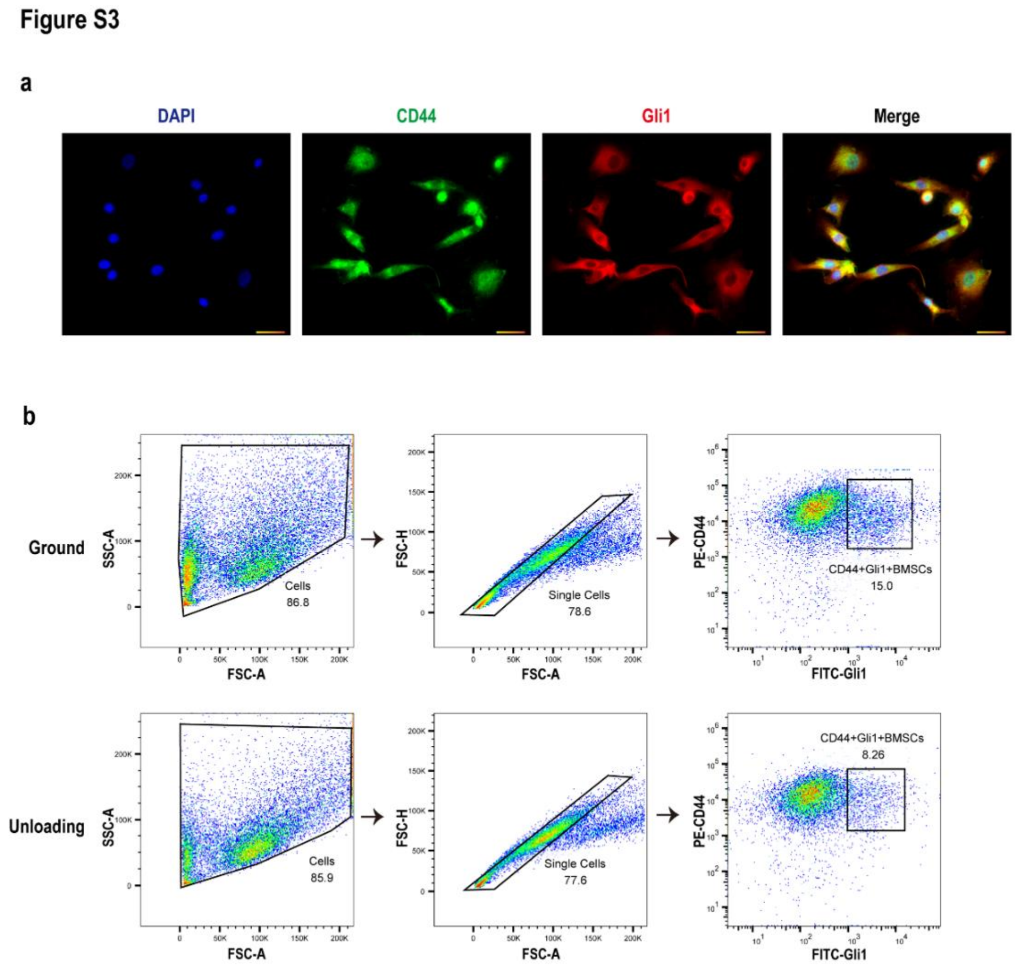

Figure S4

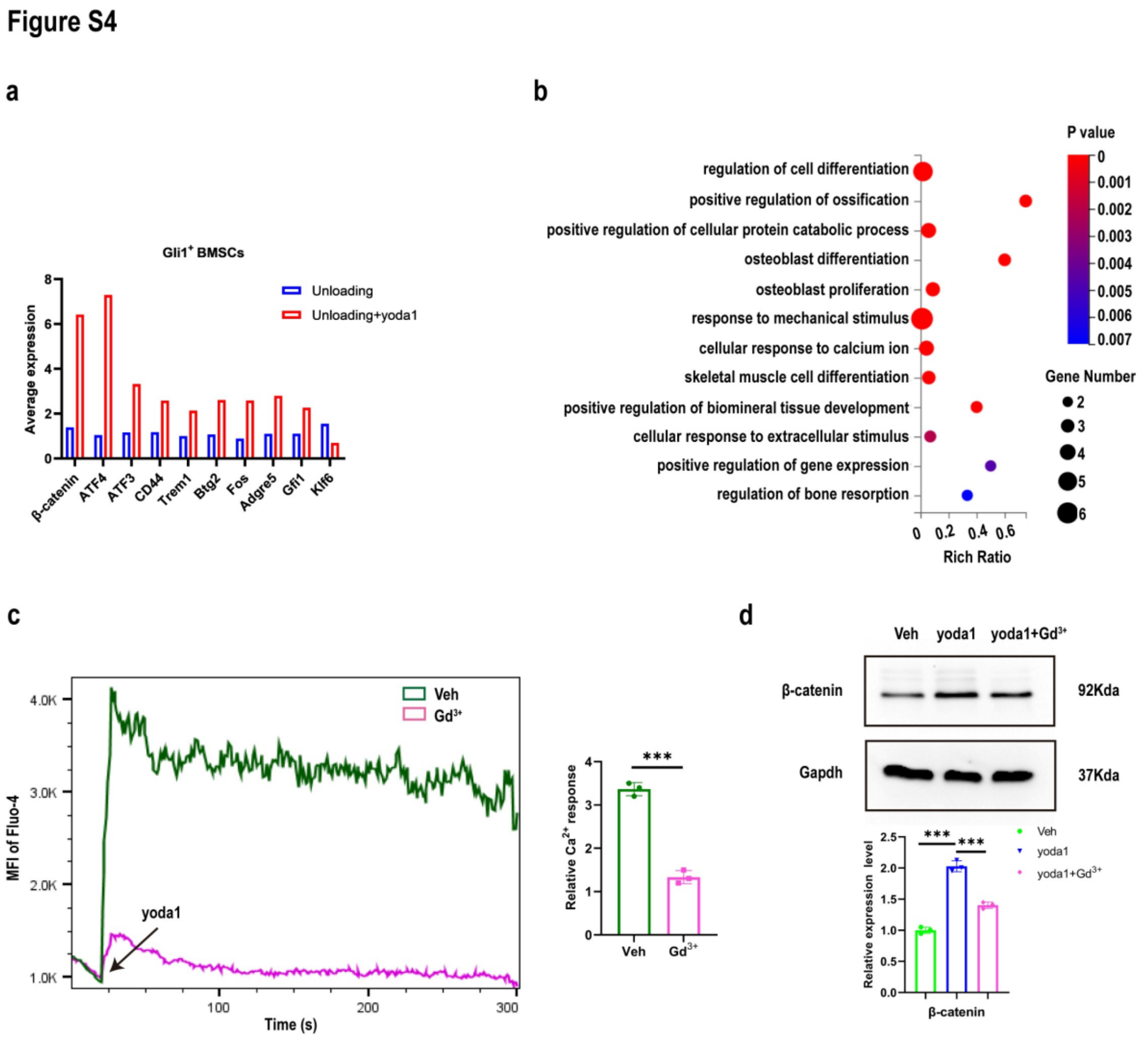

Figure S5

Figure S5

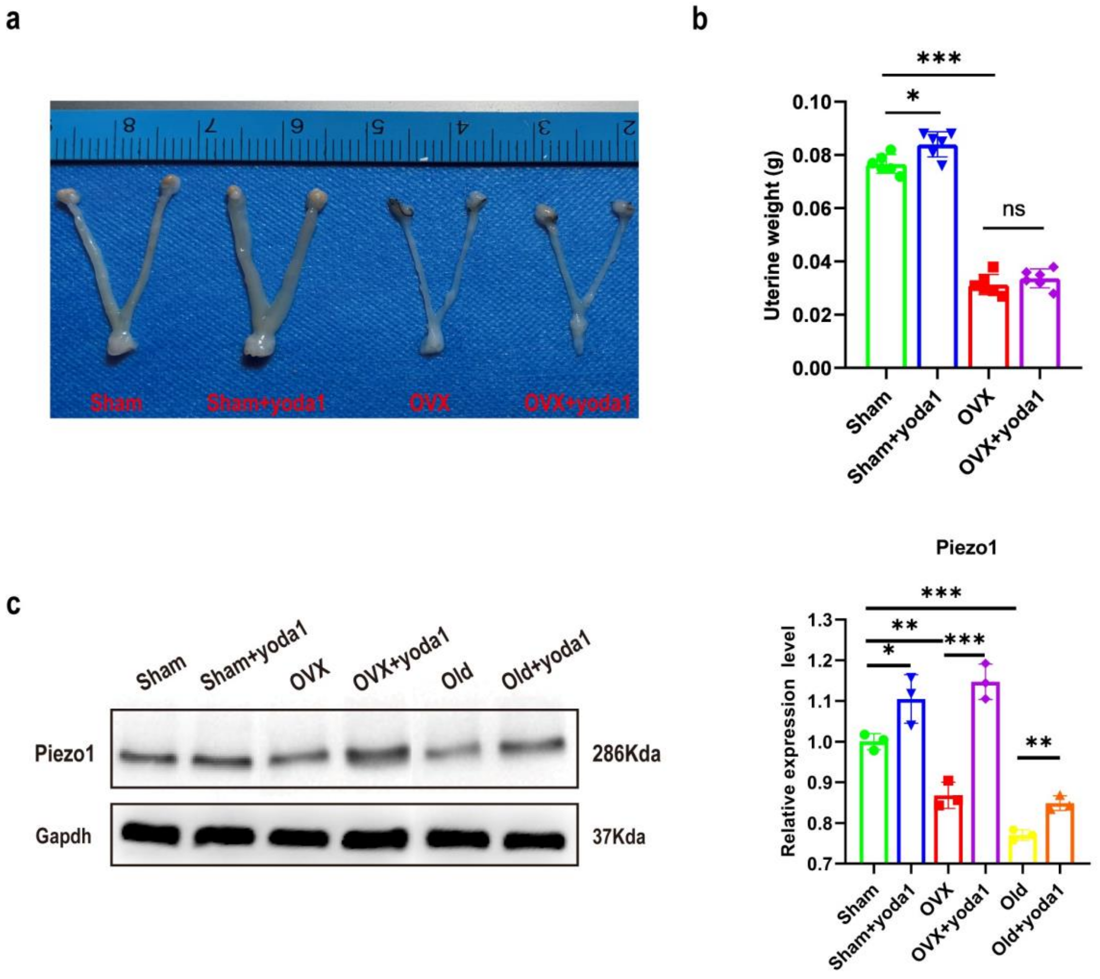

Figure S6

Figure S6

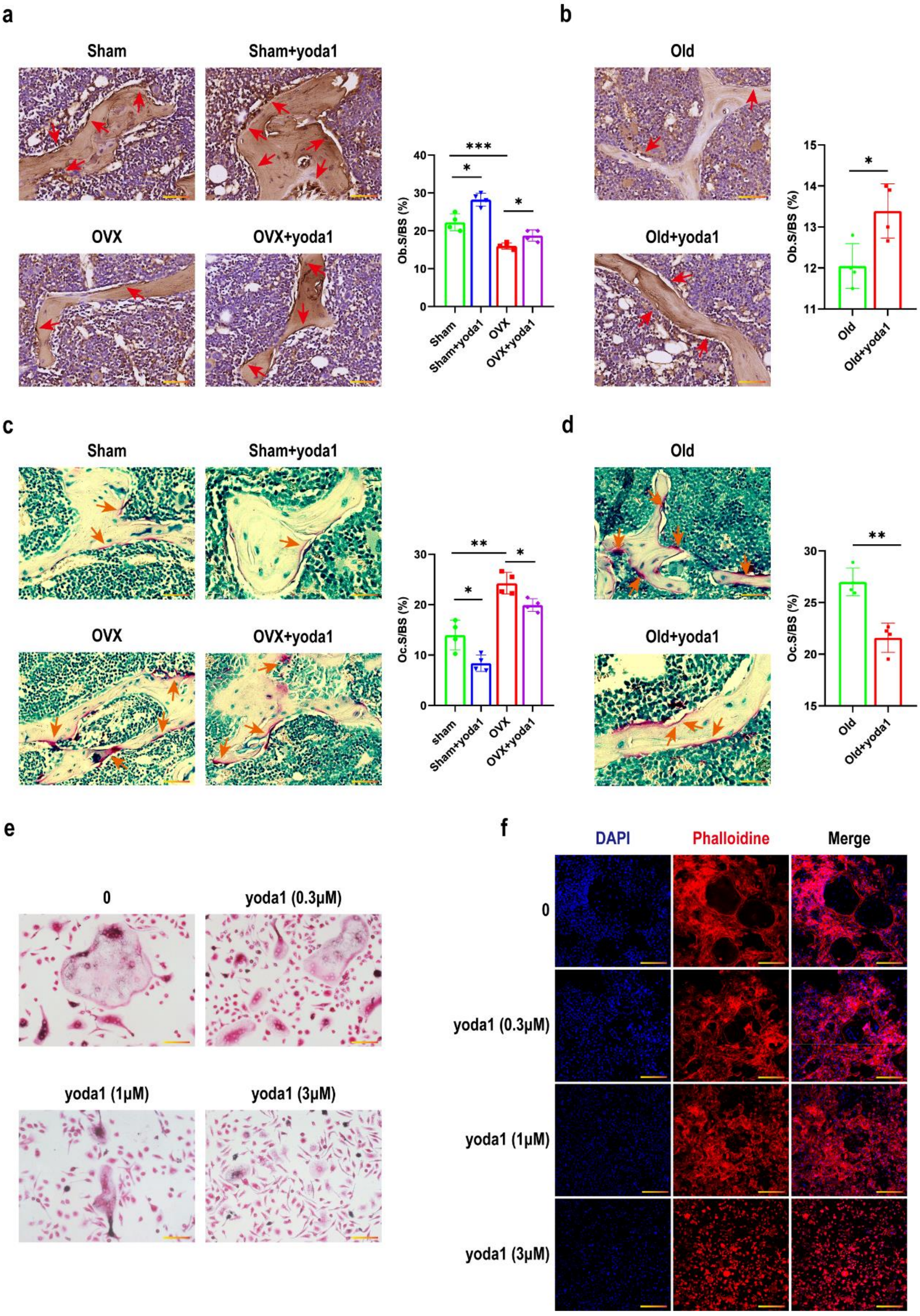
a
Sham
Sham+yoda1
OVX
OVX+yoda1
Ob.S/BS (%)
***
*
*
b
Old
Old+yoda1
Ob.S/BS (%)
*
c
Sham
Sham+yoda1
OVX
OVX+yoda1
Oc.S/BS (%)
**
*
*
sham
d
Old
Old+yoda1
Oc.S/BS (%)
**
e
0
yoda1 (0.3μM)
yoda1 (1μM)
yoda1 (3μM)
f
DAPI
Phalloidine
Merge
0
yoda1 (0.3μM)
yoda1 (1μM)
yoda1 (3μM)
